# CHALLENGES AND STRATEGIES TO IMPLEMENTING THE TRANSITIONAL CARE MODEL: LESSONS LEARNED FROM THE MIRROR-TCM TRIAL

**DOI:** 10.1093/geroni/igad104.1877

**Published:** 2023-12-21

**Authors:** Karen Hirschman, Molly McHugh, Onome Osokpo, Kathleen Mccauley, Elizabeth Shaid, Alexandra Hanlon, Mark Pauly, Mary Naylor

**Affiliations:** University of Pennsylvania, Philadelphia, Pennsylvania, United States; University of Pennsylvania, Philadelphia, Pennsylvania, United States; University of Pennsylvania, Philadelphia, Pennsylvania, United States; University of Pennsylvania, Philadelphia, Pennsylvania, United States; University of Pennsylvania, Philadelphia, Pennsylvania, United States; Virginia Tech, Blacksburg, Virginia, United States; University of Pennsylvania, Philadelphia, Pennsylvania, United States; University of Pennsylvania, Philadelphia, Pennsylvania, United States

## Abstract

The Transitional Care Model (TCM) is an advanced practice registered nurse-led, team-based, care management strategy which has repeatedly been shown to improve outcomes for older adults transitioning from hospital to home. The MIRROR-TCM randomized controlled trial was implemented at three healthcare systems (7 hospitals) in four states. Older adults, hospitalized with heart failure, chronic obstructive pulmonary disease, or pneumonia, were enrolled between September 2020 and January 2023. A parallel evaluation study of “what it takes” to implement the TCM was launched to explore the challenges and strategies necessary to implement the Model’s 10 components, especially in the context of COVID-19. A qualitative descriptive analysis of 167 meetings (clinical, community, leadership) from June 2020 (Pre-Implementation) through January 2023 (implementation/evaluation) was conducted. Codes were developed inductively over the first year of the study. The most common challenges were patient and caregiver factors (e.g., engagement, preferences, medical complexity), staffing coverage (e.g., time off, absences), and collaboration with multidisciplinary teams (e.g., poor or reluctant collaboration/communication). Common strategies to address challenges included: clarifying and adapting TCM processes, establishing an appropriate plan of care, building and engaging networks and relationships across healthcare and community settings, and trialing alternative strategies (e.g., telehealth in place of in-person visits). While some challenges were more common across sites such as patients refusing visits or no weekend coverage, strategies to resolve them tended to be site-specific. Findings provide important insights into what it takes to successfully implement complex evidence-based interventions such as the TCM.

